# Low-loss single-mode hybrid-lattice hollow-core photonic-crystal fibre

**DOI:** 10.1038/s41377-020-00457-7

**Published:** 2021-01-06

**Authors:** Foued Amrani, Jonas H. Osório, Frédéric Delahaye, Fabio Giovanardi, Luca Vincetti, Benoît Debord, Frédéric Gérôme, Fetah Benabid

**Affiliations:** 1 GPPMM Group, XLIM Institute, CNRS UMR 7252, University of Limoges, Limoges, 87060 France; 2 GLOphotonics, 123 Avenue Albert Thomas, Limoges, 87060 France; 3 Department of Engineering “Enzo Ferrari”, University of Modena and Reggio Emilia, Modena, 41125 Italy

**Keywords:** Fibre optics and optical communications, Optoelectronic devices and components

## Abstract

Remarkable recent demonstrations of ultra-low-loss inhibited-coupling (IC) hollow-core photonic-crystal fibres (HCPCFs) established them as serious candidates for next-generation long-haul fibre optics systems. A hindrance to this prospect and also to short-haul applications such as micromachining, where stable and high-quality beam delivery is needed, is the difficulty in designing and fabricating an IC-guiding fibre that combines ultra-low loss, truly robust single-modeness, and polarisation-maintaining operation. The design solutions proposed to date require a trade-off between low loss and truly single-modeness. Here, we propose a novel IC-HCPCF for achieving low-loss and effective single-mode operation. The fibre is endowed with a hybrid cladding composed of a Kagome-tubular lattice (HKT). This new concept of a microstructured cladding allows us to significantly reduce the confinement loss and, at the same time, preserve truly robust single-mode operation. Experimental results show an HKT-IC-HCPCF with a minimum loss of 1.6 dB/km at 1050 nm and a higher-order mode extinction ratio as high as 47.0 dB for a 10 m long fibre. The robustness of the fibre single-modeness is tested by moving the fibre and varying the coupling conditions. The design proposed herein opens a new route for the development of HCPCFs that combine robust ultra-low-loss transmission and single-mode beam delivery and provides new insight into IC guidance.

## Hollow-core photonic crystal fibres: Low loss with hybrid-lattice cladding

A hybrid microstructured cladding significantly reduces confinement loss and preserves single-mode operation in hollow-core photonic crystal fibres. The hybrid cladding was conceptualised and designed by Fetah Benabid of XLIM Institute, CNRS, and colleagues in France and Italy. It is made of an inner cladding of six tubes connected to an outer basket weave-like ‘Kagome’ lattice via a number of small, thin tubes. Hollow-core photonic crystal fibres surrounded by this hybrid cladding demonstrated a minimum loss figure of 1.6 decibels per kilometre at wavelengths of 1050 nanometres with robust single-mode operation in a ten-metre-long fibre. The design demonstrates the concept’s potential for single-mode waveguides and could contribute towards realising the next generation of long-haul optical fibres.

## Introduction

Due to their excellent performance as a platform for the study of fundamental physics and for addressing applied problems in photonics, hollow-core photonic-crystal fibres (HCPCFs) continue to be the subject of intense research since their theoretical proposal in 1995^1^. These interests are driven by the accomplishment of outstanding results in science, such as atom optics^2^ and gas-based nonlinear optics^3^, and in industry, such as ultra-short-pulse and high-energy laser beam delivery^4^. Furthermore, the physics of HCPCF optical guidance is still a fascinating and ongoing topic of research^5^.

Today, we distinguish two types of HCPCF: one type that guides via a photonic bandgap (PBG)^1^ and a second type that guides via the mechanism of inhibited-coupling (IC) optical guidance^6^. In PBG fibres, light is guided because the fibre microstructure is designed such that there is no cladding mode to which the core mode can couple. Conversely, in IC fibres, although there is no bandgap in the cladding frequency-effective refractive index space, the coupling of the core mode to the cladding is strongly inhibited due to a low spatial overlap between the core and cladding mode field distribution, and to a strong mismatch between the transverse spatial phases of these modes^7^. These principles provided conceptual tools to introduce the hypocycloid core contour (i.e., negative curvature) concept^8,9^, which enabled a dramatic enhancement in the light confinement of these fibres, as exemplified by IC-guiding hypocycloid core-contour Kagome lattice HCPCFs and single-ring tubular-lattice (SR-TL) HCPCFs^10^. Experimental illustrations of the negative curvature core-contour impact are the reduction in loss figures in Kagome HC-PCFs down to 8.5 dB/km at 1030 nm^11^, which is considerably lower than the dB/m loss level reported in the first Kagome fibre^12^, and the realisation of optimised SR-TL HCPCFs with a transmission loss as low as 7.7 dB/km at 780 nm^7^ and 13.8 dB/km at 539 nm^13^. Among the noteworthy conclusions of this effort is the fact that for wavelengths shorter than ~1 µm, the transmission loss is no longer limited by the cladding design. Instead, the surface-roughness-induced scattering loss (SSL) is the limiting factor. On the other hand, for wavelengths longer than ~1 µm, the confinement loss (CL), and hence the cladding design, remains the limiting factor of the IC-HCPCF transmission performance^7^. Very recently, new negative curvature cladding designs were introduced, and lower CL values than those of Kagome-HCPCFs and SR-TL HCPCFs were demonstrated. Very promising transmission loss figures were reported, as exemplified by the figures of 2 dB/km at 1512 nm for conjoined tubular cladding HC-PCFs^14^ and 0.28 dB/km at 1550 nm for nested^15^ tubular cladding HCPCFs. Table 1 summarises the measured loss figures in representative HCPCFs.

While the recent decreasing trend in the IC-HCPCF loss figures is remarkable, the current challenge in the field is to design and fabricate an IC-guiding fibre that combines ultralow loss, truly single-modeness, and polarisation-maintaining operation, especially at wavelengths much shorter than 1550 nm, e.g., around 1 µm. To illustrate the difficulty in combining only single-modeness and ultralow loss in IC-guiding fibres at ~1 µm, we recall the following. Rigorously speaking, in IC-guiding fibres, single-modeness is impossible because of the ubiquitous presence of higher-order modes in the fibre core. However, one can get closer to a truly single-mode operation if the loss extinction ratio between the lowest loss mode (typically, the core fundamental mode) and the second-lowest loss mode is sufficiently high. It was successfully achieved in six-tube SR-TL HCPCFs thanks to the adequate ratio between the core and lattice tube diameters, *D*_tubes_/*D*_core_, calculated to be 0.68, which provides effective refractive index matching between the LP_11_-like modes of the core (which are usually the main contaminating higher-order mode in the fibre modal content) and the fundamental LP_01_-like mode of the lattice tubes^16–18^. The inconvenience of this approach, however, is that the CL of the core fundamental mode is considerably high because of the small core diameter. For example, Gao et al. measured loss values of approximately 500 dB/km for wavelengths of approximately 1 µm with six-tube SR-TL HCPCFs^19^. On the other hand, the minimum loss figure reported in the literature when using IC-guiding fibres of approximately 1 µm is 2.5 dB/km, which was obtained by using a nested tubular cladding HCPCF^20^. However, the ratio between the loss values of the fundamental and LP_11_-like modes in nested fibres is typically lower than 20 dB^15^, which is considerably inferior to the ratio achieved in six-tube-lattice SR-TL HCPCFs, which can be higher than 30 dB^18^. While this ratio is sufficient to achieve effective single-mode operation under static conditions by suitable input light coupling, it becomes problematic to ensure single-modeness in conditions where the fibre is under constant motion and bending. Table 1 summarises the measured higher-order mode extinction levels in representative HCPCFs.

**Table 1:** Summary of the measured transmission loss figures and HOM (higher-order mode) extinction in representative HCPCFs

Fibre design	Minimum loss and working wavelength	Typical HOM extinction
Kagome lattice	8.5 dB/km at 1030 nm^11^	20.2 dB for 5 m^11^
Single-ring tubular lattice (SR-TL)	500 dB/km at 1000 nm	22.4 dB for 15 m (8-tube lattice)^7^
	(6-tube lattice)^19^	
	7.7 dB/km at 780 nm	
	(8-tube lattice)^7^	
	13.8 dB/km at 539 nm	
	(9-tube lattice)^13^	
Conjoined tube lattice	2 dB/km at 1512 nm^14^	>27 dB for 15 m^14^
Nested tube lattice	0.28 dB/km at 1550 nm^15^	~25 dB for 10 m^29^
	2.5 dB/km at 1000 nm^20^	
Hybrid Kagome-tubular (HKT) lattice	1.6 dB/km at 1050 nm (this work)	47.0 dB for 10 m (this work)

Here, we present the design and fabrication of a novel HCPCF structure that combines single-mode operation and ultralow CL. The cladding design exhibits a hybrid lattice made of Kagome and tubular cladding lattices. The utilisation of two IC claddings allows a decrease in CL while keeping the fibre core demarcated by a six-tube-tubular lattice for effective single-mode operation. As shown in the following, a minimum loss figure of 1.6 dB/km at 1050 nm can be experimentally achieved by using this fibre design. Moreover, the modal content of the fibre is measured by using a spatially and spectrally (*S*^2^) imaging technique^21^. The higher-order mode contributions are measured to have a maximum extinction ratio as low as 47.0 dB for a 10 m long fibre under optimised coupling conditions. Finally, the robustness of the fibre single-mode character is verified by inspecting the output mode, while the fibre is moving and by changing the coupling conditions. We believe that the fibre design proposed herein provides a new avenue for obtaining low-loss single-mode HCPCFs.

## Results

### Design rationale

**Fig. 1:**
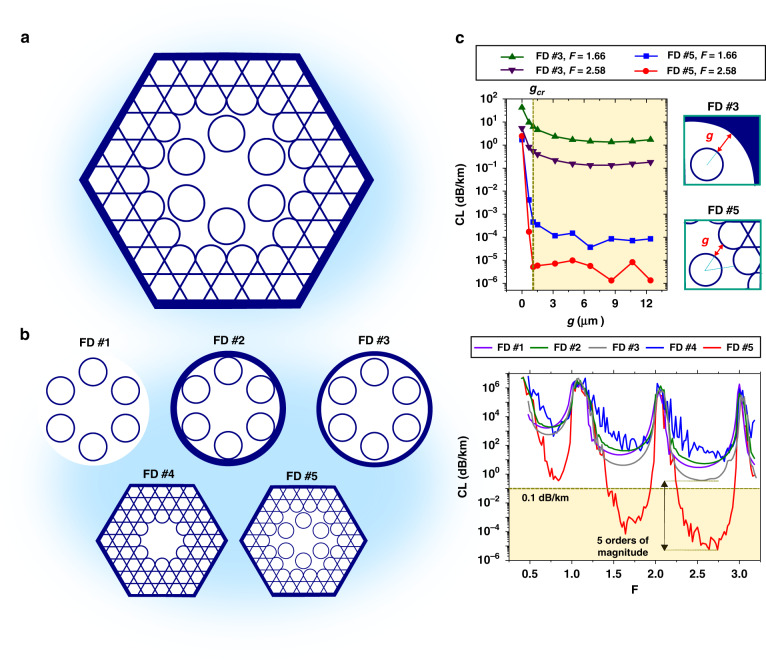
Fiber design rationale. **a** Schematic diagram of the hybrid Kagome-tubular HCPCF. **b** CL simulation results for different fibre designs (FD). FD #1: jacketless tubular lattice; FD #2: jacketed tubular lattice; FD #3: tubular lattice design with a spacing between the tubes and the silica jacket; FD #4: Kagome lattice; FD #5: hybrid lattice. **c** CL as a function of the distance (**g**) between the lattice tubes and the silica jacket (for FD #3) or between the lattice tubes and Kagome lattice (for FD #5) at selected normalised frequency values (*F*)

Figure 1 summarises the proposed fibre structure, the hybrid Kagome-tubular lattice (HKT) HCPCF, and its design rationale. Figure 1a shows the fibre transverse geometrical microstructure. It is endowed with a two-lattice cladding. The inner cladding consists of a lattice of six tubes, which demarks the fibre core. The six-tube lattice is chosen to attain effective single-mode operation via resonant filtering of the LP_11_ mode^16–18^. Additionally, this inner cladding stands out because of the absence of connection nodes, which favours the IC guidance^7^. The outer cladding of the HKT-HCPCF comprises a Kagome lattice structure in which the tubular lattice is embedded. As will be demonstrated in the following, the association of two IC claddings significantly enhances the light confinement in the core and reduces the fibre CL expressively.

We start our analysis by considering an ideal version of the HKT-HCPCF in which the tubular and Kagome lattices are not physically connected. Even though the ideal structure is unrealistic, it allows exploring the potential of the novel design proposed herein to provide ultralow loss, and to deepen our understanding of the enhancement of the confinement capabilities when combining two IC cladding architectures. Moreover, the ideal version of the fibre acts as a pedagogical and pre-design tool that reveals the most important design elements and physical aspects of the proposed fibre architecture, namely, the importance of having a suitable spacing between the inner and outer claddings, the effect of the number of Kagome cladding layers, and the location of the leakage channels in the structure. The presentation of a realisable version of the HKT-HCPCF will follow the study of the ideal version of it.

The fibre design is driven by the guiding principles of the IC guidance mechanism^6,7^. The inner cladding is made with six isolated tubes because doing so ensures single-mode operation and is void of connecting nodes. The surrounding cladding is made by using another IC cladding, i.e., the Kagome lattice, to reduce tunnelling leakage. Furthermore, the CL induced by the nodes and sharp bends in the outer cladding is minimised, because of their larger distance from the fibre core.

Figure 1b shows the cross-sections of the fibre designs (FDs), we study herein (*lhs*) and their respective simulated CL as a function of the normalised frequency values, $F = \left( {2t/\lambda } \right)\sqrt {n_{\rm{g}}^2 - 1}$ (*rhs*). Here, *t* and *n*_g_ represent the thickness and refractive index of the cladding structure, respectively, and *λ* is the wavelength. In the CL calculation, all the fibres are taken to have the same core inner diameter (35 µm), *t* (1100 nm), and *n*_g_(1.45). The first design (FD #1, purple line) consists of a jacketless 6-tube lattice structure whose minimum CL values are approximately 1650 dB/km, 20 dB/km, and 3 dB/km in the fundamental, first-order, and second-order transmission bands, respectively. The second design (FD #2, green line) reproduces a 6-tube lattice SR-TL HCPCF. The results show that jacketing the suspended tubes impacts the CL spectrum mildly, with an increase in the above loss figures to approximately 1900 dB/km, 40 dB/km, and 5.5 dB/km, respectively. FD #3 (grey line), in turn, is a modified version of the tubular lattice design with tubes that are fully isolated. The spacing between the lattice tubes and the silica jacket is set to 1.59 µm. In this idealised design, the existence of a gap between the tubes and the jacket affects the CL values, which are found to be lower than those calculated for FD #1 and FD #2.

The blue line in Fig. 1b shows the CL for a Kagome lattice HCPCF, identified as FD #4. For this design, the minimum CL figures are approximately 440 dB/km, 50 dB/km, and 30 dB/km in the fundamental, first-order, and second-order transmission bands, respectively. We note for this fibre the oscillating structure in the CL spectrum due to connecting struts or nodes^6^, which can either locally increase or decrease the CL compared to FD #1 and FD #2.

The HKT-HCPCF we propose herein is identified in Fig. 1b as FD #5. The red line in Fig. 1b shows the ideal case of this structure. In this situation, there is no physical connection between the tubular and Kagome lattices, and the spacing between the lattices is set to 1.59 µm. Although this is not a feasible fibre design, it communicates the potential of this novel design to achieve impressively low CL values—as low as 0.35 dB/km, 7 × 10^−5^ dB/km and 8.6 × 10^−6^ dB/km for wavelengths within the fundamental, first-order, and second-order transmission bands, respectively. It is noteworthy that the two latter values for this ideal structure are well below the current attenuation level of solid-core fibres, represented by the yellow dashed line in Fig. 1b^22^. Additionally, there is an expressive difference of five orders of magnitude between the CL of FD #5 and those calculated for the other designs, as emphasised by the black arrow in Fig. 1b. Particularly, a comparison between the results of FD #3 and FD #5 allows ascribing the CL reduction to the replacement of the homogeneous silica jacket by the Kagome microstructure.

Figure 1c shows the evolution, with respect to the spacing between the inner and outer claddings in FD #3 and FD #5 (*g*, see inset of Fig. 1c), of the minimum CL in the 1st and 2nd transmission bands. The results for FD #5 readily show that, for a significant drop in CL, *g* must be larger than a critical value *g*_cr_. When *g* is varied from 0.68 to 1.51 µm, the CL drops from 4.1 × 10^−3^ dB/km to 3.5 × 10^−4^ dB/km at *F* = 1.66 and from 1.7 × 10^−4^ dB/km to 5.8 × 10^−6^ dB/km at *F* = 2.58. The case where *g* = 0 is also shown in Fig. 1c. For this case, the CL values are much higher, as high as 1.7 dB/km at *F* = 1.66 and as high as 2.4 dB/km at *F* = 2.58. In these simulations, the alteration in the *g* values is achieved by adequately enlarging the Kagome lattice pitch so that the core diameter (*D*_core_ = 35 µm), the distance between the cladding tubes (*δ* = 6.5 µm), and the thickness of the tubes and Kagome lattices (*t*_tubes_ = *t*_kago_ = 1100 nm) can be maintained. Based on the data presented in Fig. 1c, the critical *g* value (*g*_cr_) is found to be approximately 1.5 µm for attaining the dramatic reduction in the CL in the HKT-HCPCF design compared to that in the usual Kagome and tubular fibre designs. It is noteworthy that the results of our numerical study (not shown) also indicate that the azimuthal position of the inner cladding relative to the outer cladding affects the CL. The results for FD #3, in turn, also teach that increasing *g* entails a reduction in the CL values. However, as the confining power of the homogeneous silica jacket is low, the decreasing trend of the CL values is not as extreme as in FD #5.

Moreover, the concept of CL reduction by associating two IC lattices can be expanded to other IC cladding architectures, such as those with inner claddings made with nested tubes or conjoined tubes (see Supplemental Material for further information). The CL obtained with both the nested tube and conjoined tube inner claddings is comparable with that of the tubular lattice, but they exhibit a structured loss spectrum because of the presence of connecting nodes. Furthermore, it is worth-mentioning that it is particularly challenging to fulfil the required phase matching between the LP_11_ core mode and LP_01_ inner cladding air modes for single-modeness when using nested tube lattices. Additionally, extending the hybrid design to different outer cladding structures other than the IC structures is a plausible direction. For example, one can propose a different configuration in which the outer Kagome lattice is replaced with a PBG cladding. Indeed, the concept of the association of two claddings to enhance light confinement remains valid. However, further investigation is needed if this approach is chosen since care must be taken in the design and fabrication of the interface between the inner and outer claddings, and in the choice of their respective structural dimensions.

**Fig. 2:**
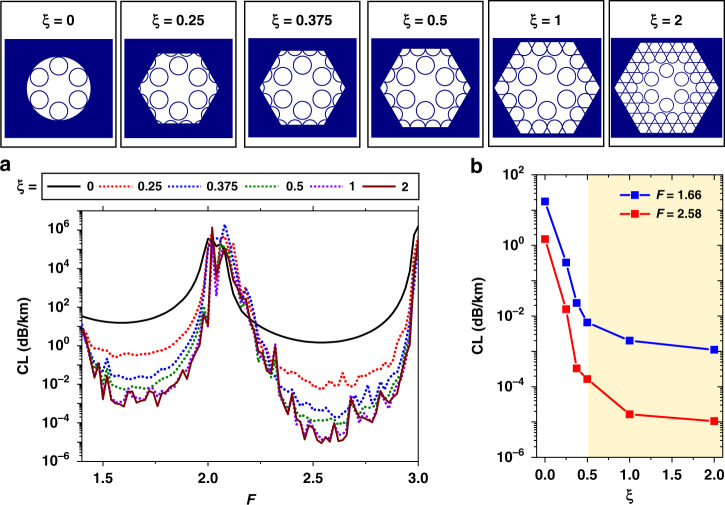
Study on the effect of adding the Kagome lattice around the tubular lattice in the HKT-HCPCF design. **a** CL simulation results for fibre designs with different *ξ* values (see text for the definition). **b** CL values for two selected normalised frequency (*F*) values

**Fig. 3:**
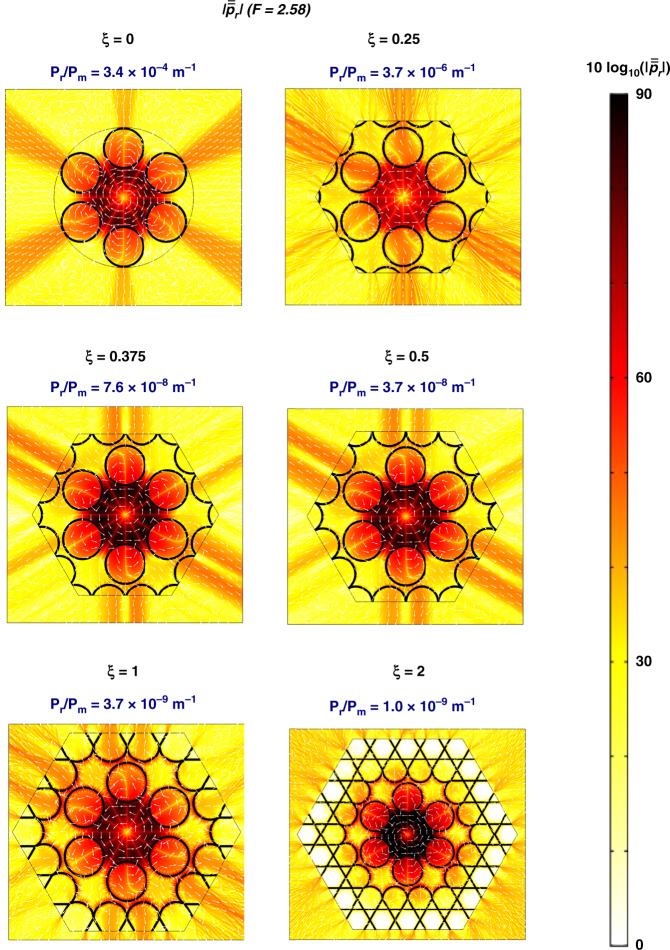
Modulus of the normalised radial component of the Poynting vector for representative values of *ξ* at *F* = 2.58, and the respective $P_{\mathrm{r}}/P_{\mathrm{m}}$ values. Adding the Kagome structure around the tubes that form the tubular lattice allows minimising the strength of the leaking channels of the structure. The colour scale refers to $10\log _{10}\left( {\left| {\overline{\overline p} _{\rm{r}}} \right|} \right)$

To provide physical insight into the increase in confinement power in the HKT lattice cladding, we recall that, in contrast to PBG fibres, whose loss figures monotonically decrease with an increasing number of cladding layers^23^, the CL in Kagome fibres does not display the same behaviour. Instead, the minimum CL in Kagome fibres is reached for an optimum number of layers, which stems from a trade-off between the cladding structure confining power and the growth in the cladding density of photonic states^6^. Indeed, although adding cladding layers improves the confinement of the mode in the core, it concurrently creates additional cladding modes to which the core mode is weakly coupled, thus entailing further CL. Therefore, the optimum CL in Kagome lattices is accomplished by adequately considering the compromise between the number of photonic states in the cladding and the confining power of the structure. This feature is investigated and corroborated in a systematic study summarised in Figs. 2 and 3.

Figure 2 shows the CL when considering a 6-tube lattice structure and sequentially adding the Kagome structure around the tubular cladding layer by layer. For simplicity, we define a parameter *ξ* as the ratio between the thickness of the considered outer cladding in the simulations and the Kagome cladding pitch. In our analysis, *ξ* is varied from 0 (no Kagome cladding) to 2 (Kagome lattice composed of two rings of tubes), and the calculated CL values are presented in Fig. 2a. Figure 2b shows the CL values for two representative normalised frequency values (*F* = 1.66 and *F* = 2.58). The effect of adding the Kagome cladding on the CL drop rate is extremely drastic between *ξ* = 0 and *ξ* = 0.5 and gentler for *ξ* > 1. At *F* = 1.66, the CL is calculated to be 17.4 dB/km when *ξ* = 0 and 6.5 × 10^−3^ dB/km when *ξ* = 0.5. At *F* = 2.58, the CL accounts for 1.5 dB/km when *ξ* = 0 and 1.6 × 10^−4^ dB/km when *ξ* = 0.5. When *ξ* = 1 and *ξ* = 2, a further decrease, but at a slower rate, is observed in the CL values. At *F* = 1.66, the CL is calculated as 1.1 × 10^−3^ dB/km when *ξ* = 2. At *F* = 2.58, the CL is calculated as 1.0 × 10^−5^ dB/km when *ξ* = 2. Here, it is worth-noting that having a Kagome cladding formed by more than two rings of tubes (i.e., having *ξ* > 2) entails a marginal decrease in the CL^24^. In the simulations shown in Fig. 2, the core diameter (*D*_core_ = 33.5 µm), the distance between the cladding tubes (*δ* = 4.67 µm), the distance between the tubular and Kagome lattices (*g* = 1.59 µm), the Kagome cladding pitch (19.46 µm), the curvature parameter (*b* = 1, which indicates circular-shaped boundary arcs), and the tube and Kagome lattice thicknesses (*t*_tubes_ = *t*_kago_ = 1100 nm) were kept constant.

Figure 3 presents the transverse leakage maps for different *ξ* (the fibre parameters are the same as in Fig. 2). The colour diagrams show that at *F* = 2.58 and for representative values of *ξ*, the modulus of the normalised radial component of the Poynting vector, $\overline{\overline p} _{\rm{r}} = \frac{{{\rm{Re}}\left( {\vec p \cdot \hat r} \right)}}{{P_{\rm{r}}}}$, where $\vec p = \frac{1}{2}\overrightarrow E \times \overrightarrow {H^ \ast }$ is the Poynting vector (with $\overrightarrow E$ and $\overrightarrow H$ representing the electric and magnetic fields, respectively), $\hat r$ is the radial unit vector, and $P_{\rm{r}} = {\oint} {\vec p \cdot \hat r{\rm{d}}l}$ is the leaked power per unit length. Indeed, $\overline{\overline p} _{\rm{r}}$ has been proved to be a useful tool in examining the leakage dynamics in IC fibres^7^. In the Supplemental Material, we expatiate the relationship between $\overline{\overline p} _{\rm{r}}$ and the fibre CL and provide a further discussion on the normalisation procedure. Additionally, we show in Fig. 3 the attenuation values calculated as $\frac{{P_{\rm{r}}}}{{P_{\rm{m}}}}$, where *P*_m_ is the real part of the mode power, with $P_{\rm{m}} = {\rm{Re}}\left( {\mathop {\iint}\nolimits_{{\rm{S}}_\infty } {\vec p \cdot \hat z{\rm{d}}S} } \right)$.

Consistent with the results published by Debord et al.^7^, the distance between the cladding tubes in the fibre design studied here (*δ* = 4.67 µm) entails the main channel for the core fundamental mode leakage to be the direction through the lattice tubes (instead of the direction through the gap between the cladding tubes; see the results for *ξ* = 0). For *ξ* > 0, one sees that the presence of the Kagome lattice around the tubular lattice minimises the power flux through this leaking channel of the tubular lattice structure. This leakage reduction increases with increasing *ξ* for the values considered here. It is noteworthy that the main leaking channels for the Kagome lattice are azimuthally shifted from the direction through the inner lattice tubes. Additionally, the vortex-like dynamics of the radial component of the Poynting vector contour lines inside the fibre structure are remarkable. This interesting behaviour, also observed in other works^25,26^, calls for further exploration of the transverse energy flow in HCPCFs.

**Fig. 4:**
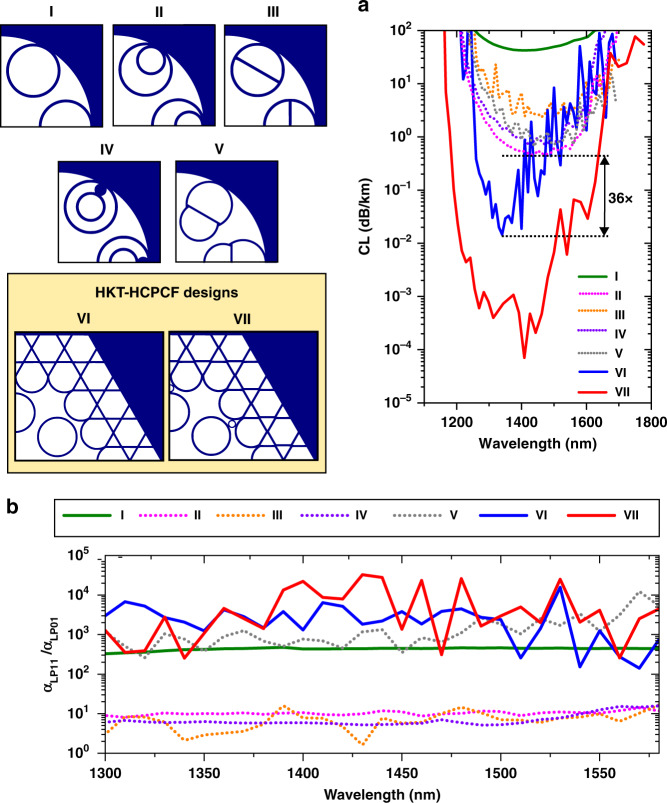
Hybrid Kagome-tubular HCPCF versus reported HCPCF designs. **a** Fundamental mode CL simulation results for the (I) tubular, (II) nested tubular, (III) straight-bar tubular, (IV) nested-rod tubular, (V) conjoined tubes, (VI) ideal hybrid and (VII) hybrid with supporting tube fibre designs. **b** The ratio between the LP_11_ mode and fundamental mode CL values (α_LP11_/α_LP01_) in the studied fibre designs

Figure 4 presents the HKT lattice design potential for offering excellent CL and HOM extinction levels among the representative IC-HCPCF designs explored in the literature. Figure 4a shows a comparative plot between the CL of the fundamental mode in the SR-TL HCPCF (I, green line), nested (II, pink dashed line), straight-bar (III, orange dashed line), nested-rod (IV, purple dashed line), conjoined tubes (V, grey dashed line), and hybrid design proposed herein (VI). The data represent fibres with a core diameter of 30.5 µm and lattice tubes with an outer diameter and thickness of 22.1 µm and 1.1 µm, respectively. In design V, the tubes in the second ring layer have a diameter of 26.4 µm, and in designs II and IV, the nested tubes have a diameter of 12.17 µm. The Kagome lattice pitch in designs VI and VII is set as 18 µm. These parameters are considered to allow a direct comparison to the data recently published by Habib et al.^27^.

Figure 4a shows that fibre designs II, III, IV, and V allow decreasing the CL values of SR-TL HCPCFs (I) by approximately two orders of magnitude. However, their CL remains within the range of ~1–3 dB/km, with the nested design (II) having the minimum value of 0.5 dB/km for the fibre parameters considered here. In contrast, the ideal hybrid fibre structure (VI, red line) offers a dramatic CL reduction. The CL values drop to 7 × 10^−5^ dB/km around 1410 nm. In this simulation, the distance between the tubular and Kagome lattices was set as 2.04 µm.

While the ideal HKT-HCPCF design has suspended inner cladding tubes (therefore, it is not feasible), it is a structure of important academic interest for demonstrating the concept of the association of two IC claddings towards the reduction in the CL, as well as the potential of the HKT-HCPCF to provide impressive low-loss figures. Taking this into account, we show in Fig. 4 (VII, blue line) a realisable version of the HKT-HCPCF. In this fibre design, the cladding tubes are connected to the Kagome lattice via the utilisation of thin tubes. Indeed, simulations show that the thinner the connection tubes are, the lower the CL values will be. Here, we choose connecting tubes with a thickness of 640 nm (i.e., 58% of the cladding struts and tube thickness) as a reasonable value considering the HKT-HCPCF fabrication. The results show that while the addition of the connecting tubes causes the CL to increase, the minimum CL of 1.46 × 10^−2^ dB/km at approximately 1340 nm is 36 times lower than the lowest CL figure achieved with the other fibre designs between 1260 and 1400 nm—as emphasised by the black arrow in Fig. 4a.

Additionally, we evaluate in Fig. 4b the ratio between the CL of LP_11_-like modes and the CL of the fundamental mode (α_LP11_/α_LP01_) to assess the potential of the fibre designs for single-mode operation. We distinguish two design groups: the first group (I, V, VI, and VII) with α_LP11_/α_LP01_ in the range of 3 × 10^2^ to 3 × 10^4^ and the second group (II, III, and IV) with a much lower ratio, in the range of 2–10. This difference is readily explained by the fibre design LP_11_ mode resonant filtering capability^16–18^. Here, the hybrid fibre design is the only one that attains a better compromise in the binomial CL and HOM extinction. Considering the results for the hybrid fibre design, if one assumes a situation in which 99% of the power is coupled to the fundamental mode and 1% is coupled to the LP_11_ mode at the fibre input, an HOM extinction ratio higher than 40 dB can be estimated for a 10 m long fibre.

### Fibre fabrication and loss measurement

**Fig. 5:**
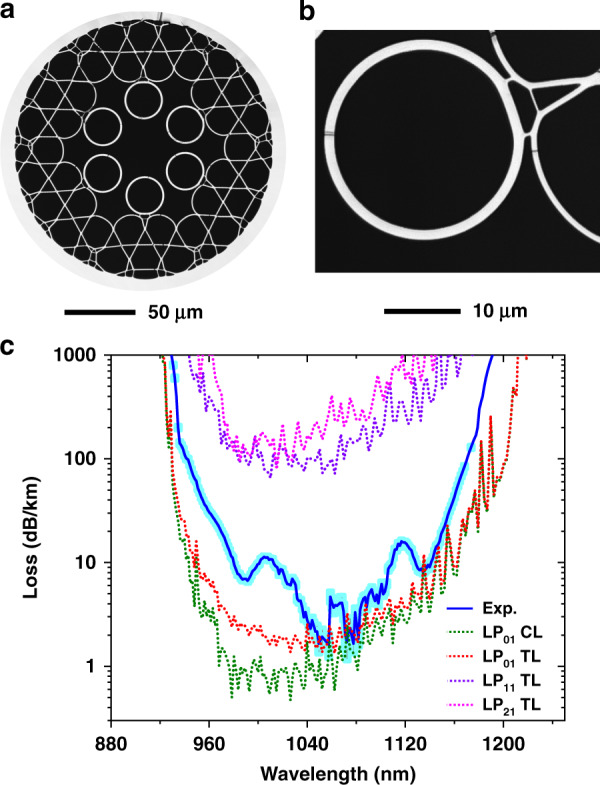
Fabricated Hybrid Kagome-tubular HCPCF. **a** HKT-HCPCF cross-section and **b** enlarged view of the supporting tube region. **c** Cutback measurement results (blue curve), simulated CL and TL for the fundamental mode (green and red dotted lines, respectively), and the simulated TL for representative higher-order modes (purple and pink dotted lines)

Due to the potential of the HKT-HCPCF shown by the simulations, we endeavoured to obtain such a fibre experimentally. The cross-section of the fabricated fibre is presented in Fig. 5a, which was obtained by using the stack-and-draw technique. The tubes that form the tubular lattice have a thickness of 1.27 μm and an inner diameter of 23.0 μm. The fibre core has a 37.1 μm diameter. During fabrication, the tubes and core sizes were optimised to achieve *D*_tubes_/*D*_core_ = 0.62. This value is close to the optimum value of *D*_tubes_/*D*_core_ = 0.68, which is calculated to be the one that provides the optimum coupling between the LP_11_ mode guided in the core and the fundamental mode guided in the lattice tubes^18^. The Kagome lattice struts have a uniform thickness of 720 nm, and the supporting tubes have a thickness of 370 nm, both within a 50 nm variation (see Fig. 5b for an enlarged view of the supporting tube region). In the current development stage, the fabricated fibre lengths are typically between 100 and 200 m.

Figure 5c presents the loss of the HKT-HCPCF (blue curve), which was measured from a cutback measurement using 120 and 4 m long fibre pieces. A minimum loss value of 1.6 ± 0.4 dB/km was measured at 1050 nm. Additionally, the simulated CL and TL (total loss) of the fundamental mode (green dotted curve for the CL and red dotted curve for the TL) and the TL of the representative HOM (namely, the LP_11_-like and LP_21_-like modes—purple and pink dotted lines, respectively) are shown in Fig. 5c. Here, it is worth clarifying that the CL values were calculated by considering the real fibre cross-section. In turn, the TL was calculated by using ${\rm{TL}} = {\rm{CL}} + {\rm{SSL}}$, where SSL is the surface scattering loss, which was estimated by using ${\rm{SSL}} = \eta F_{{\rm{cc}}}\left( {\frac{{\lambda _0}}{\lambda }} \right)^3$, where *η* is a constant linked to the surface roughness height, *F*_cc_ is the core mode overlap with the core contour, and *λ*_0_ is a constant that allows calibrating the SSL formula^28^ (in Fig. 5c, *η* = 0.25 × 10^−2^ and *λ*_0_ = 1700 nm). Good agreement is seen between the simulated TL of the fundamental mode and the experimentally measured loss. The HOM loss is found to be approximately two orders of magnitude higher than the loss of the fundamental mode, in good agreement with the predicted losses (see Fig. 4b).

### Modal content measurement and further characterisation results

**Fig. 6:**
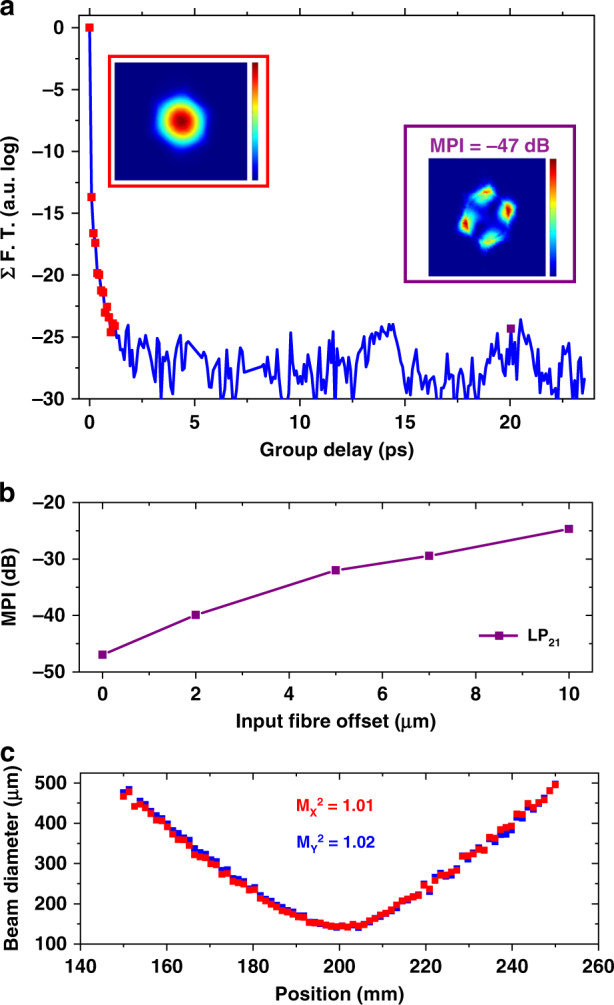
HKT-HCPCF modal content characterisation. **a** *S*^2^ measurement results for a 10 m long fibre. **b** MPI as a function of the input fibre offset. **c** *M*^2^ measurement results

*S*^2^ measurements^21^ were performed to quantitatively account for the modal content of the fibre. Figure 6a shows the *S*^2^ trace for a 10 m long HKT-HCPCF. The latter shows that no contribution of the LP_11_ mode was detected (which shows that the 6-tube tubular-lattice fibre design filtered it). A contribution of an LP_21_-like mode was detected with an MPI (multipath interference) value as low as −47.0 dB. Table 1 shows the HOM suppression values accounted for by the *S*^2^ measurements in the IC-HCPCFs. The values reported for the tubular, nested tube, and conjoined tube designs range from 27 to 22.4 dB for fibres with lengths between 10 and 15 m. The HKT-HCPCF reported herein, therefore, allows obtaining an improved effective single-mode operation in IC-HCPCFs.

To show the fibre single-mode operation robustness, we conducted *S*^2^ measurements by applying an offset in the input fibre position relative to the laser beam (for a fibre with a length of 10 m). The MPI values for the LP_21_ mode are plotted in Fig. 6b as a function of the input fibre offset (the zero offset represents the optimum light transmission). It is observed that when the coupling efficiency to the LP_21_ mode grows as the input fibre offset becomes larger, the MPI values for the LP_21_ mode increase from −47.0 to −24.7 dB when the input fibre offset is increased from 0 to 10 μm. It is noteworthy that even for this large offset in the fibre input position, the HOM contributions have a maximum MPI value of −24.7 dB in the proposed HKT-HCPCF and that no LP_11_ contribution can be observed. Furthermore, Fig. 6c presents a typical *M*^2^ measurement (performed at 1030 nm using the ISO standard, Metrolux LPM 200) for the HKT-HCPCF. The *M*^2^ values were measured to be 1.01 and 1.02 for the *x*-axes and *y*-axes, respectively. Moreover, the fibre PER was tested. A maximum value of 21 dB was measured for a 10 m long fibre at 1030 nm. Additionally, the fibre bend loss was characterised. The measurements showed that at 1100 nm, the bend loss values were approximately 0.06 dB/turn when the curvature radius was 20 cm.

**Fig. 7:**
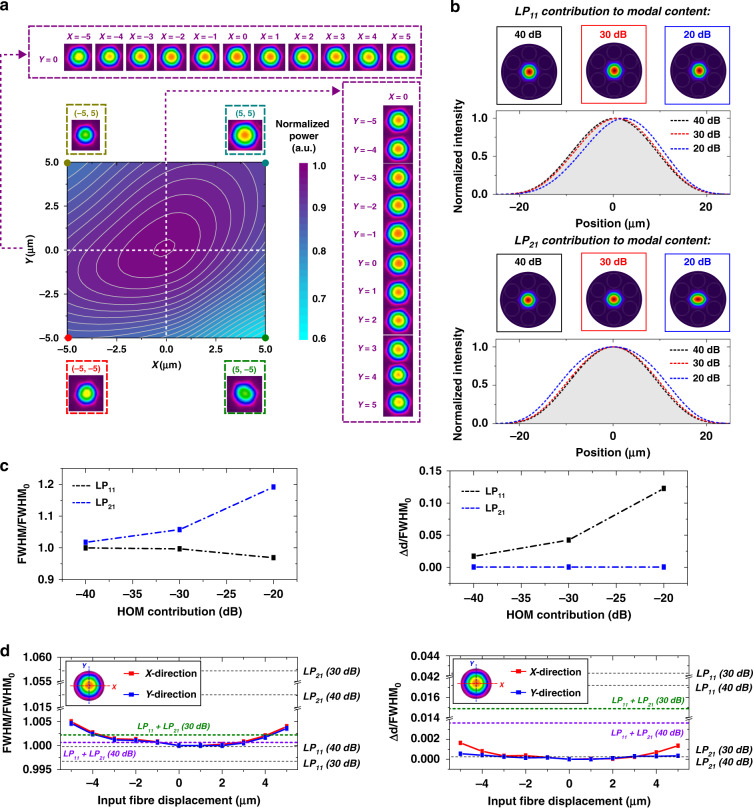
Single modedness robustness. **a** Normalised power and near-field profiles at the fibre output for different input conditions at 1064 nm. **b** Intensity mode profiles considering a fibre modal content dominated by the fundamental mode and with different HOM levels added to the modal content. **c** Simulated normalised beam full width at half maximum (FWHM/FHWM_0_) and centroid variation (Δ*d*/FHWM_0_) for different HOM contributions to the fibre modal content. **d** Measured FWHM/FHWM_0_ and Δ*d*/FHWM_0_ of the output beam as the input fibre position was scanned along the horizontal direction, i.e., *Y* = 0 in **a**. Horizontal lines represent the simulated FWHM/FHWM_0_ and Δ*d*/FHWM_0_ values. In the superposition cases, we assume equally weighted combinations of the LP_11_ and LP_21_ modes contributing to the modal content at extents of 30 and 40 dB

Finally, the robustness of the fibre single-mode operation against laser beam misalignment with the fibre core was tested by examining the reconstructed near-field profile under different coupling conditions. To do this, we used a laser at 1064 nm and measured the power and the output near the field profile as the input fibre position was scanned along the horizontal and vertical axes. The scan spans 10 µm along both axes, i.e., Δ*x* = Δ*y* = ±5 µm. The origin corresponds to the output power maximum, measured to be 90% of the input power. For each displacement, the fibre output power and near-field profile were recorded. Figure 7a shows a colour map of the normalised power distribution, with the selected near-field profiles shown in the insets (along the horizontal axis, along the vertical axis and at extreme positions). It is shown that over the 10 × 10 µm^2^ area, the near-field profile remained fundamental-like, and the transmitted power remained higher than 60% of the maximum. It is worthnoting that the colour map is not perfectly symmetric due to imperfect light coupling at the fibre input.

As additional indicators of the fibre effective single-mode operation, we investigate in Fig. 7 the effect of the HOM contribution to the full width at half maximum (FWHM) and the centroid position of the output beam. Figure 7b shows the calculated intensity mode profiles, dominated by the fundamental mode, when assuming different intensity contributions from the LP_11_ and LP_21_ modes to the modal content (namely, 40, 30, and 20 dB). The plots in Fig. 7b present the intensity profiles along the horizontal axis for the cases mentioned above (the shaded areas represent the intensity profile of the fundamental mode). The most pronounced effect of adding the LP_11_ mode to the fibre modal content is shifting the beam centroid. On the other hand, the most noticeable effect of including the LP_21_ mode on the fibre modal content is changing the beam FWHM. These observations can be verified in Fig. 7c, where the normalised FWHM (FWHM/FHWM_0_, where FWHM_0_ is the full width at half maximum of the fundamental mode) and the centroid shift normalised by the FHWM_0_ (Δ*d*/FHWM_0_) of the beams are plotted as a function of the HOM contribution to the modal content. The results show that the LP_11_ and LP_21_ contributions corresponding to a power extinction of 30 dB led to an ~5% relative change in the FWHM and centroid position. Figure 7d presents graphs of the normalised FWHM and centroid variation of the output beam as the HKT-HCPCF input position was scanned along the *X*-axis (*Y* = 0 in Fig. 7a). The results show the measured FWHM/FHWM_0_ and Δ*d*/FHWM_0_ to be less than 0.5% and less than 0.2%, respectively, over the whole scan range. Additionally, the figure shows the HOM contribution to the FWHM/FHWM_0_ and Δ*d*/FHWM_0_ variations for different LP_11_ and LP_21_ modal combinations (dashed horizontal lines). The results show that, independent of the LP_11_ and LP_21_ combination, the HOM contribution to the modal content remained less than 30 dB over the whole scanned area. Finally, it is noteworthy that this range in the FWHM/FHWM_0_ and Δ*d*/FHWM_0_ variation was not increased when the fibre was coiled and shaken during the recording.

## Discussion

The results reported herein demonstrate that the association of two IC claddings can significantly reduce the CL and modal content in HCPCFs. Specifically, we used an inner cladding with six suspended tube rings to ensure single-mode operation by resonantly filtering the lowest loss HOM and an outer cladding with a Kagome lattice for CL enhancement. The results show that when we surround the inner tubes with a Kagome lattice without allowing them to touch (i.e., using the ideal HKT-HCPCF), a drop in CL by 5 orders of magnitude relative to a typical tubular or Kagome cladding HCPCF and to the most representative reported HCPCF alternative designs can be achieved (a minimum of 8.6 × 10^−6^ dB/km was obtained with the explored fibre parameters). When considering the realisable version of the HKT-HCPCF design, the simulations revealed that a minimum loss figure of 1.42 × 10^−2^ dB/km at approximately 1340 nm can be achieved.

By making use of this concept, we developed and fabricated a hybrid cladding IC-HCPCF whereby the inner and outer cladding layers are connected via smaller and thinner tubes to keep the fibre cladding modal spectrum as close as possible to the ideal cladding design. The experimental characterisation of the fibre showed a minimum loss of 1.6 dB/km at 1050 nm, which is the lowest loss figure obtained at ~1 µm with HCPCFs, and a maximum extinction ratio of −47.0 dB for the higher-order modes measured in an *S*^2^ measurement using a 10 m long fibre, which is the highest extinction ratio reported in HCPCFs. We found that the single-mode operation of the reported fibre is very resilient against fibre motion and laser beam misalignment. This property is particularly important for high-power laser beam delivery in laser micromachining applications.

Improvements to the current HKT-HCPCF to achieve lower transmission should consider achieving better control of the shapes and sizes of the connecting tubes between the tubular and Kagome lattices during the fibre draw. If better control of this aspect can be obtained, a transmission loss reduction by more than one order of magnitude is expected. Moreover, further modifications to the fibre design, such as replacing some of the six tubes with others exhibiting different confining powers, will allow exploration of its potential as a polarisation-maintaining waveguide. The present results will contribute to worldwide endeavours to explore IC-HCPCFs as candidates for next-generation long-haul optical fibres, and will deepen our knowledge of the guidance mechanisms in these fibres.

## Materials and methods

### Fibre fabrication

The HKT-HCPCF was obtained by using the stack-and-draw technique in a two-step process. The first step involves the preform assembly and cane drawing. The second step involves drawing the canes to the fibre dimensions. During the fibre drawing procedure, independent pressurisation was applied to the Kagome lattice, tubular lattice, and core regions to attain the required geometrical sizes.

### Cutback measurement

In the cutback measurements, light from a supercontinuum light source was coupled to the fibre, and an optical spectrum analyser measured the transmitted signal. For each fibre length (120 and 4 m), the transmitted spectrum was measured for three independent fibre cleavages.

### *S*^2^ measurement

The *S*^2^ measurement setup encompassed a tuneable laser with a wavelength range between 1030 and 1070 nm (10 pm resolution) and a camera with its image acquisition routine controlled by a computer. The mode profiles and the corresponding multipath interference values were calculated from the fibre output images acquired during laser wavelength sweeping^21^. Before the *S*^2^ measurements, the fibre transmission was optimised to obtain the maximum transmitted power. The MPI values shown in Fig. 6b were obtained from *S*^2^ measurements made by offsetting the fibre input from its optimum position.

### PER measurement

In the PER measurements, light from a laser emitting at 1030 nm was launched into the fibre core, and the optical power emerging from the ports of a polarisation beam splitter was measured.

## Supplementary information

Supplemental Material

## Data Availability

The data that support the findings of this study are available from the corresponding author upon reasonable request.
