# Supplementary material for: Low-loss single-mode hybrid-lattice hollow-core photonic-crystal fibre
Source: Light Sci Appl. 2021 Jan 6;10:7. doi: 10.1038/s41377-020-00457-7 (PMC7788080; doi:10.1038/s41377-020-00457-7)
Supplement: Supplementary file 1 — Supplemental Material [file 41377_2020_457_MOESM1_ESM.docx]

**Supplemental material for**

**Low-loss single-mode hybrid-lattice hollow-core photonic crystal fiber**

Foued Amrani,^1, 2^ Jonas H. Osório,^1^ Frédéric Delahaye,^1, 2^ Fabio Giovanardi,^3^ Luca Vincetti,^3^ Benoît Debord,^1, 2^ Frédéric Gérôme,^1, 2^ Fetah Benabid^1, 2^

*^1^GPPMM Group, XLIM Institute, CNRS UMR 7252, University of Limoges, Limoges, 87060, France*

*^2^GLOphotonics, 123 Avenue Albert Thomas, Limoges, 87060, France*

*^3^Departament of Engineering “Enzo Ferrari”, University of Modena and Reggio Emilia, Modena, 41125, Italy*

1. Association of different inhibited-coupling claddings: additional simulations

In the following, we delve into different inhibited-coupling cladding architectures. Fig. S1 presents the configurations we investigate herein. Fig. S1a presents the dispersion curves of the designs. It is worth observing that the dispersion curves overlap each other. It allows us to compare the CL figures shown in Fig. S1b directly.

We start by presenting a typical tubular lattice fiber (I) and a modified version of it (II). In II, we consider a spacing (2 µm) between the lattice tubes and the silica cladding. The observation of the CL results allows concluding that the existence of a gap between the lattice tubes and the jacket causes the CL values to decrease. As extensively studied in the main text of the manuscript, embedding the tubular lattice inside a Kagome one (III) entails a dramatic reduction in the CL figures.

Additionally, we conducted an analogous systematic study for a first ring made with nested tubes (IV, V, VI) and conjoined tubes designs (VII, VIII, IX). Fiber designs IV and VII in Fig. S1 represent the typical nested and conjoined tubes structures. Designs V and VIII, in turn, refer to modified nested and conjoined tubes designs where a spacing was considered in-between the lattice tubes and the silica jacket. Similarly to the tubular design results, the CL values for designs V and VIII are found to be lower than the ones calculated for the designs IV and VII. Notably, for fiber design V, additional resonance peaks are seen in the CL spectrum (compared to IV). They are due to the existence, in V, of structural nodes in the connecting region between the tubes. These connecting regions support modes with low azimuthal order to which the core mode can be coupled.

Moreover, in Fig. S1, we investigate the combination of nested tubes (VI) and Kagome, and conjoined tubes and Kagome lattices (IX). The examination of Fig. S1b data allows recognizing that the association of two IC claddings entails a reduction of the CL in all the considered cases. However, similarly to V, the existence of connecting nodes in VI and IX entails a considerable number of resonance peaks in the CL spectrum of these fiber designs. It is worth recalling that, in these simulations, we consider the ideal case of the hybrid fiber designs, *i.e.*, when there is no physical connection between the inner and outer claddings.


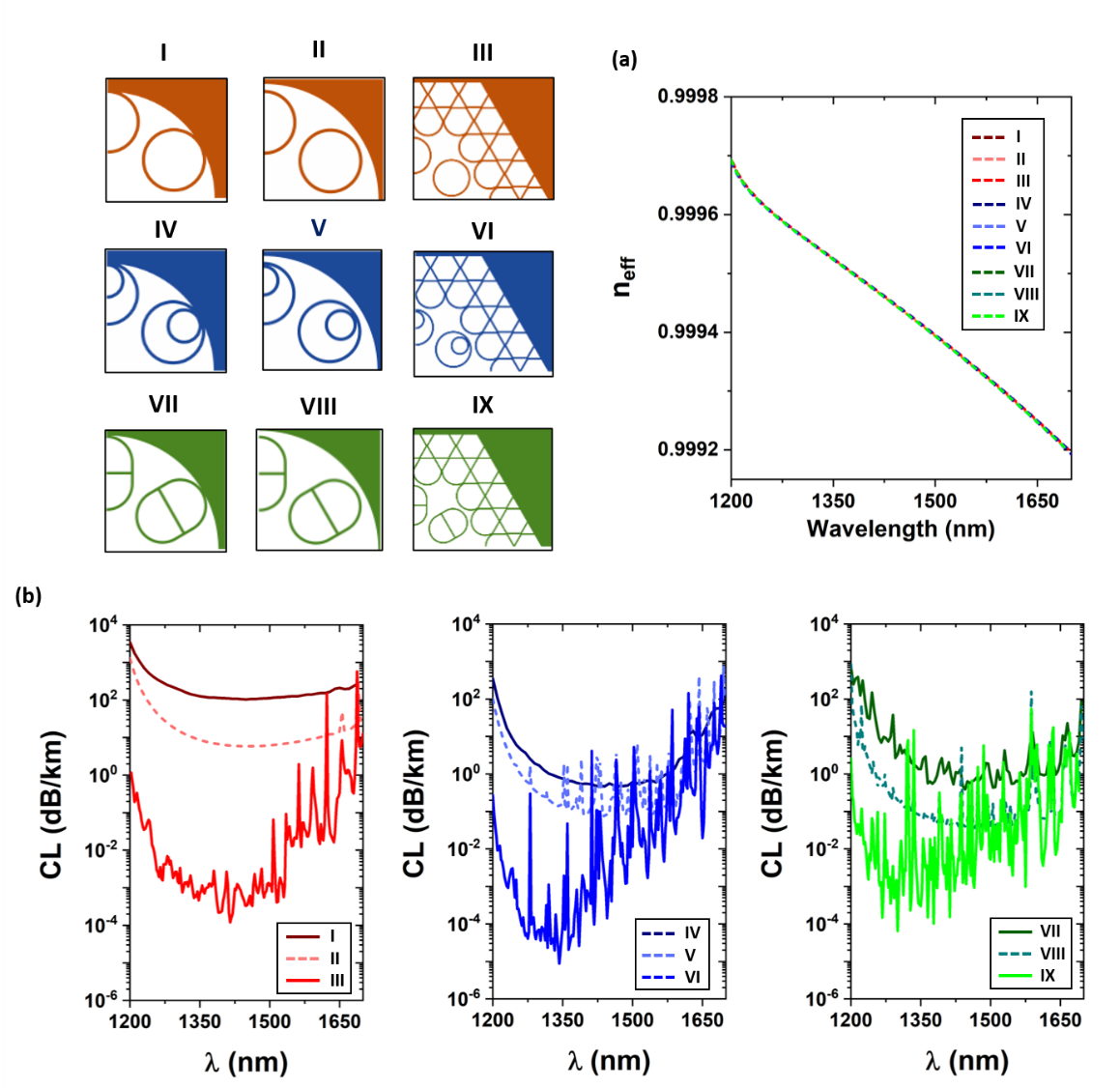


**Fig. S1.** (a) Dispersion and (b) CL curves of the analyzed fiber designs. I: tubular fiber design; II: tubular fiber design with a gap between the lattice tubes and the silica jacket; III: Hybrid Kagome-tubular lattice design; IV: nested tubes fiber design; V: nested tubes fiber design with a gap between the lattice tubes and the silica jacket; VI: Hybrid nested tubes-Kagome lattice design; VII: conjoined tubes design; VIII: conjoined tubes design with a gap between the lattice tubes and the silica jacket; IX: Hybrid conjoined tubes-Kagome lattice design.

To identify the origin of the high loss peaks in the simulated CL spectra, Fig. S2 presents the color maps of the real and imaginary parts of the transverse field (*E_t_*) of the fundamental mode in the considered hybrid-lattice designs at selected wavelengths. The plots of *Im*(*E_t_*) allows observing that the high loss peaks are due to couplings into the inner cladding structures. For wavelengths at which there are no expressive high loss peaks, couplings to the Kagome lattice still exist but are much weaker. One sees that *Im*(*E_t_*) is typically 6 decades lower than *Re*(*E_t_*) at wavelengths presenting low loss whereas, at wavelengths experiencing high loss, *Im*(*E_t_*) is typically 2 decades lower than *Re*(*E_t_*).


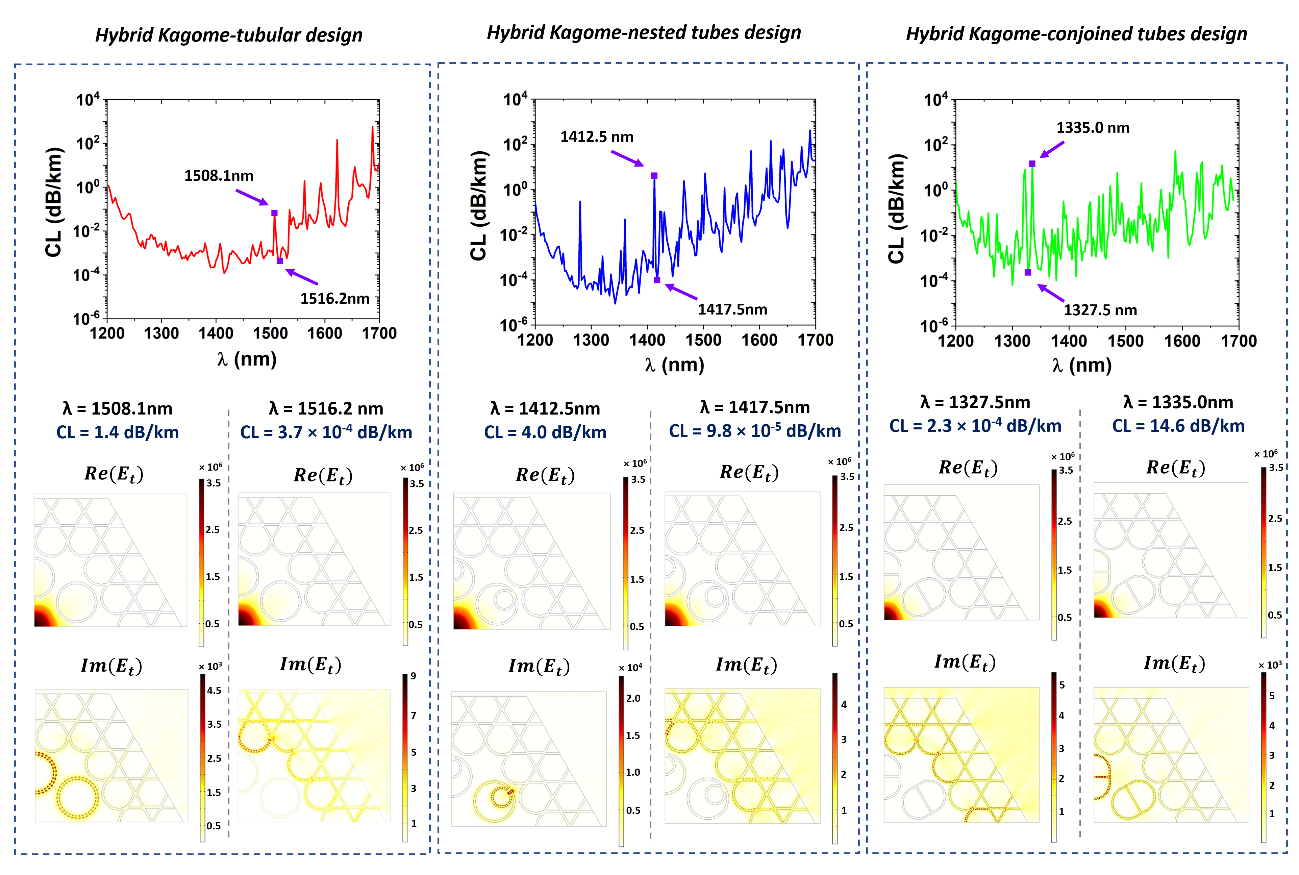


**Fig. S2.** Study on the origin of the high loss peaks in hybrid lattices composed of tubular and Kagome, nested tubes and Kagome, and conjoined tubes and Kagome lattices. High loss peaks arise from resonances in the inner cladding elements.

1. Additional information on the Poynting vector plots

The plots in Fig. 3 in the manuscript main text aim to highlight the leaking channels in the studied fiber designs. In the following, we revisit the analysis, available in Debord *et al.*^1^, where the rationale of using the transverse Poynting vector plots to study the leakage in IC fibers has been described.

We start by recalling that the radial component of the Poynting vector, $p_{r}=\vec{p}\cdot\hat{r}$, gives the density of power along the radial direction, where $\vec{p}=\frac{1}{2}\vec{E}\times\vec{H^{*}}$ is the Poynting vector, with $\vec{E}$ and $\vec{H}$ representing the electric and magnetic fields, respectively, and $\hat{r}$ is the radial unit vector. In turn, the integration of $p_{r}$ along a circle $l$ surrounding the fiber microstructure, *i.e.*, $P_{r}=\oint\vec{p}\cdot\hat{r}dl$, provides the leaked power per unit length. As shown in Debord *et al.*^1^, the confinement loss (CL) can be obtained by calculating $CL=\frac{P_{r}}{P_{m}}$, where $P_{m}$ is the mode power, $P_{m}=Re\left( \iint_{S_{\infty}} \vec{p}\cdot\hat{z}dS \right)$.

Therefore, as $P_{r}=P_{m} CL$, the normalized radial component of the Poynting vector plotted in Fig. 3 in the main text, $p_{r}=\frac{Re\left( \vec{p}\cdot\hat{r} \right)}{P_{r}}$, directly correlates with the CL of the studied structures. Besides, it gives additional information on the leakage mechanism in the considered fiber designs as it allows identifying the leaking channels of the claddings.

Finally, it is worth mentioning that, although we have used the leaked power per unit length, $P_{r}$, to normalize the radial component of the Poynting vector in our plots, there are other possible approaches for the normalization of the leaky modes, *e.g.*, the use of an integral over the longitudinal component of the Poynting vector^2-4^. Indeed, as the leaking is minimal in the fiber designs studied in the manuscript, our computations show that the difference between the distinct normalization routines is negligible. In the following, we present details on our normalization procedure.

In Snyder and Love^5^, the normalization coefficient for a mode of a nonabsorbing waveguide is defined as $|P_{c}|$, where $P_{c}=\int_{s_{\infty}} \frac{1}{2}\vec{E}\times\vec{H^{*}}\cdot\hat{z}dS$. Since in a nonabsorbing waveguide with a proper choice of the origin of the reference system both $\vec{E}$ and $\vec{H}$ are real vectors, then $P_{c}=\left| P_{c} \right|=P_{m}$, where $P_{m}$ is $P_{m}=Re\left( \int_{s_{\infty}} \frac{1}{2}\vec{E}\times\vec{H^{*}}\cdot\hat{z}dS \right)$.

Similarly, the normalization coefficient for a leaky mode is defined as $N_{c}$, where $N_{c}=\int_{s_{\infty}} \frac{1}{2}\vec{E}\times\vec{H}\cdot\hat{z}dS$^5^. Thus, if the mode is not leaky, both $\vec{E}$ and $\vec{H}$ are real vectors and, therefore, $P_{c}=\left| P_{c} \right|=P_{m}=N_{c}$. The imaginary parts of $P_{c}$ and $N_{c}$ are linked with the leakage strength in a leaky mode. In Table S1, we show the values of $\frac{P_{c}}{P_{m}}$ and $\frac{N_{c}}{P_{m}}$ at F = 2.58 (normalized frequency) for different values of $\xi$, the ratio between the thickness of the outer cladding considered in the simulations and the Kagome cladding pitch (as it was defined in the manuscript main text). We observe that the imaginary parts are, at least, 6 orders of magnitudes lower than the real parts. Thus the difference between a normalization coefficient and the other is negligible.

**Table S1.** Values of $\frac{P_{c}}{P_{m}}$ and $\frac{N_{c}}{P_{m}}$ at F = 2.58 for different values of $\xi$.

| $\xi$ | $\frac{P_{c}}{P_{m}}$ at F = 2.58 | $\frac{N_{c}}{P_{m}}$ at F = 2.58 |
| --- | --- | --- |
| 0 | $1-i\cdot5.8511170198744820E-11$ | $0.9999999615820307-i\cdot1.2586304051837839E-6$ |
| 0.25 | $1-i\cdot1.4487515110477376E-13$ | 0.9999999999099537-$i\cdot$3.784745116563714E-9 |
| 0.50 | $1-i\cdot3.0313897596858253E-15$ | 0.9999999999986803-$i\cdot$6.453674428840455E-11 |
| 1.00 | $1-i\cdot1.8193717814770378E-16$ | 0.9999999999997848-$i\cdot$7.2670537637157135E-12 |
| 2.00 | $1-i\cdot4.473235746772701E-17$ | 0.9999999999994319-$i\cdot$4.671160047919838E-12 |

References

1. Debord, B. *et al.* “Ultralow transmission loss in inhibited-coupling guiding hollow fibers,” Optica **4**, 209-217 (2017).
2. Li, G., Martinj de Sterke, C. & Palomba, S., “General analytic expression and numerical approach for the Kerr nonlinear coefficient of optical waveguides,” Opt. Lett. 42, 1329-1352 (2017).
3. Elsawy, M. M. R. & Renversez, “Exact calculation of the nonlinear characteristics of 2D isotropic and anisotropic waveguides,” Opt. Lett. 43, 2446-2449 (2018).
4. Upendar, S., Allayarov, I., Schmidt, M. A. & Weiss, T., “Analytical mode normalization and resonant state expansion for bound and leaky modes in optical fibers – an efficient tool to model transverse disorder,” Opt. Express 26, 355079 (2018).
5. Snyder, A. W. & Love, J. D., Optical Waveguide Theory (Chapman & Hall, 1983).
